# Detection of NOTCH1 mutations in paraffin samples in patients with potentially malignant lesions

**DOI:** 10.4317/jced.60293

**Published:** 2023-03-01

**Authors:** Maria Baus-Domínguez, Raquel Gómez-Díaz, Guiomar Martín-Lozano, Fernando Iglesias-Martín, José-Luis Gutiérrez-Pérez, Daniel Torres-Lagares, María-Ángeles Serrera-Figallo, Aida Gutiérrez-Corrales

**Affiliations:** 1 Department of Oral Surgery, Faculty of Dentistry, University of Seville, Seville, Spain; 2 Institute of Biomedicine of Seville, Seville, Spain; 3 Oral and Maxillofacial Unit, Virgen del Rocio Hospital, Seville, Spain. Oral Surgery Department, Faculty of Dentistry, University of Seville, Seville, Spain

## Abstract

**Background:**

On certain occasions, oral cancer is preceded by potentially malignant lesions. The degree of dysplasia in Guinea pigs attempts to determine the risk of developing a malignant lesion. The search for genetic mutations, biomarkers, as a more truthful and reproducible diagnostic tool, tries to fill the gaps in the anatomopathological study. In this line, the present retrospective case-control study is based on the detection of known mutations of the NOTCH1 gene in biopsied samples of potentially malignant lesions from 22 patients who attend the Oral and Maxillofacial Surgery service of the Virgen del Rocío University Hospital.

**Material and Methods:**

DNA extraction after dewaxing of the samples using the Minikit QIAamp DNA FFPE tissue extraction kit with extraction kit (reference 56404) of QIAGEN. Subsequently, with the DNA obtained, 4 amplification reactions were carried out using enzyme polymerase. Before sequencing the samples, they were purified with the ExoSAP-IT for PCR product cleaning kit of the INVITROGEN brand. Finally, to detect somatic mutations in NOTCH1, TaqMan Mutation Detection Assays was used and for the analysis of mutations we worked with the Mutation Detector software.

**Results:**

The mutation for NOTCH1 is not detected, the studied sample does not present the mutation, or it is below the limits of detection of the software.

**Conclusions:**

In the clinical setting of the sample, the NOTCH1 mutation seems to be not very frequent, although NOTCH1 has been described as a gene related to oral cancer in other geographical settings.

** Key words:**Oral cancer, NOTCH1, mutations.

## Introduction

As has already been described on numerous occasions. Today, oral cancer is the 16th most common malignancy (>300.000 cases annually) according to the 2020 Global Cancer Observatory data (GLOBOCAN). About, 90-95% of oral cancers are squamous cell carcinoma (OSCC). This type of cancer is more common in men over 40 years of age; its multifactorial etiology and is associated, as is well known, with the consumption of tobacco (in any form) being the most important risk factor for the development of oral cancer and alcohol, in frequent and abundant consumption, and also having a synergistic effect with tobacco (1-5). Other factors related to the development of oral cancer, according to the American Society of Clinical Oncology and the World Health Organization (WHO), include prolonged exposure to the sun, especially in cases of lip carcinoma; Human Papillomavirus (HPV) infections; insufficient oral hygiene in conjunction with continuous mechanical trauma and in patients who smoke and drink alcohol are considered a risk factor; as well as poor diet and nutrition with a low consumption of fruits and vegetables.

The number of oral cancers has increased considerably in the elderly population, in large part due to the increased longevity. Therefore, it is estimated that in the next 20 years there will be an overall increase of 66.2% in the number of new cases in this population (2).

Similarly, despite numerous technological advances, oral cancer remains a disease with a high mortality and morbidity rate, as treatment remains a major challenge and survival rates have not experienced a significant increase in recent decades (1,2,6).

These data highlight the importance of knowing how to recognize the characteristics of oral cancer to develop new prevention and early diagnosis actions.

However, even though we know the characteristics in terms of prevalence and the multiple risk factors associated with this type of lesions, the diagnosis of oral cancer in its initial stages remains complex, as it usually does not present symptoms and the changes that can be observed at the level of the oral mucosa become so tenuous that it is difficult to detect it in a routine oral examination (2,7).

In certain cases, oral cancer is preceded by a series of lesions now called ‘Oral Potentially Malignant Disorders (OPMD)’ (WHO Classification of Head and Neck Tumors, 4th edition, 2017), due to their potential for malignant transformation that they may experience on certain occasions, although not all (7-9). Among the most common potentially malignant lesions that can precede the development of oral cancer are leukoplakia, erythroplakia, and leukoerythroplakia. All are purely clinical terms that designate a white lesion, a red lesion, or a white and red lesion, respectively, that does not peel off when scraped and cannot be diagnosed like any other age (3,9). Other OPDMs according to the WHO 2017 classification are oral submucous fibrosis, dyskeratosis congenita, smoking-free tobacco keratosis, reverse smoker palatal changes, chronic candidiasis, oral lip ligand, diskoid lupus erythematosus, syphilitic glossitis and actuinic cheilitis. Therefore, the true diagnosis will come, as always, from the histological examination of the biopsy.

However, predicting the risk of malignant transformation of these lesions remains a great challenge, as although biopsy and its pathological examination constitute the current gold standard for predicting this risk, some authors warn of a prognostic value limited by subjectivity and lack of safe reproducibility (7), since the fact that some lesions present a low degree of dysplasia is not exempt from experiencing an evolution toward carcinoma (7,8).

Since cancer is known to be the result of a series of processes indispensable for the maintenance of malignancy, resistance to cell death, continuous proliferation, evasion of tumor suppressor genes, angiogenesis, and activation of invasion and metastasis stand out. And, since these processes are mediated by a complex and extensive network of molecular signaling pathways (10), many professionals are working on the search for a method using valid and reproducible molecular biomarkers that can predict more objectively the malignant transformation (4) that an injury can experience to achieve an improvement not only in diagnosis, if not, in prognosis, treatment, and follow-up of patients who have suffered and/or suffer from OSCC. For example, loss of p53 function associated with proliferation and evasion of growth suppressors or dysregulation of NOTCH1 pathways that help to enable replicative immunity and prevent cell differentiation (10).

NOTCH proteins are single-step transmembrane receptors that regulate cell fate decisions during stem cell development, maintenance, proliferation, and apoptosis (3-6,10,11).

Exome sequencing in 2011 first reported mutations in NOTCH1 and showed that it was the second most frequently mutated gene in head and neck squamous cell carcinomas (HNSCC) after TP53 (1,4,6,8,9,11). NOTCH1 is physiologically expressed in the basal cells of the oral squamous epithelium and its expression is inhibited in oral cancer and oral epithelial dysplasia (10).

INACTIVATE NOTCH1 inactivating mutations are present in 11-19% of HNSCC, suggesting that NOTCH1 may behave as a tumor suppressor in contrast to its proto-oncogenic role in other cancers (1,3-10,12,13). On the contrary, activation of the NOTCH1 signaling pathway can promote the progression of oral squamous cell carcinoma (OSCC) (7,10).

Despite the numerous roles that a mutation in the NOTCH1 gene can play in the etiopathogenesis of oral cancer, this study aims to search for NOTCH1 mutations in biopsied lesions diagnosed with potentially malignant disorders and / or oral carcinoma in a first cohort of 22 patients, in order to increase the sample in the future to find more exhaustively whether NOTCH1 could function as a molecular biomarker and, therefore, as a predictor of the risk of developing neoplasia in patients from the south of Spain who present lesions diagnosed as potentially malignant disorders of the oral cavity.

## Material and Methods

-Type of study

Retrospective case-control study approved by the Ethics Committee of the Virgen del Rocío Hospital - Exp PI-0081-2016, carried out in patients of the Virgen del Rocío University Hospital in Seville who present prospectively malignant lesions biopsy candidates.

This is a descriptive observational study in which the only invasive procedure, in addition to biopsy to diagnose the lesion, was the extraction of a small amount of blood in conjunction with a examination of the oral cavity.

All patients included in the study were fully informed about the nature of the study, and patients or the person responsible gave their express consent based on the direct patient benefits of the investigation.

-Selection of patients and study groups

The study population consists of patients of both sexes who come to the Oral and Maxillofacial Surgery Unit of the Virgen del Rocío University Hospital in Seville to assess potentially malignant oral lesions.

The patients had to meet each of the inclusion criteria and none of the exclusion criteria.

The inclusion criteria are as follows.

• Patients over 18 years of age 

• Patients with potentially malignant oral lesions (leukoplakias, erythroplakias, or leukoerythroplakias) in the oral mucosa (lateral edge of the tongue, anterior floor of the mouth, and jugal mucosa) biopsy candidates.

•Patients who agree to participate in the study (informed consent)

The exclusivity criteria are as follows:

• Patients with vascular malformations

• Patients with a history of oral mucosal surgery were explored

• Patients with poor general condition that prevents their exploration and sampling

• Patients with intellectual inability to understand and/or sign informed consent

If, after a conventional oral clinical examination based on inspection and palpation, the physician considered that the lesion should be biopsied, it was proposed that the patient participate in the study.

-Biopsy

The biopsy was performed by the first surgeon to perform conventional scans of all patients according to the initial decision he had made.

All biopsies are performed according to the usual protocol, containing part of the healthy mucosa and part of the lesion mucosa.

All biopsies underwent a routine pathological study and were evaluated according to the WHO classification (no dysplasia, mild dysplasia, moderate dysplasia, severe dysplasia, carcinoma in situ, and infiltrating carcinoma) by two pathologists who are experts in head and neck pathology.

All biopsies were obtained during 2015. After the result of the biopsies was obtained, the tissue was included in paraffin for the genetic study of the same. However, DNA quantification does not start until November 2016 (Table 1).

**Table 1:**
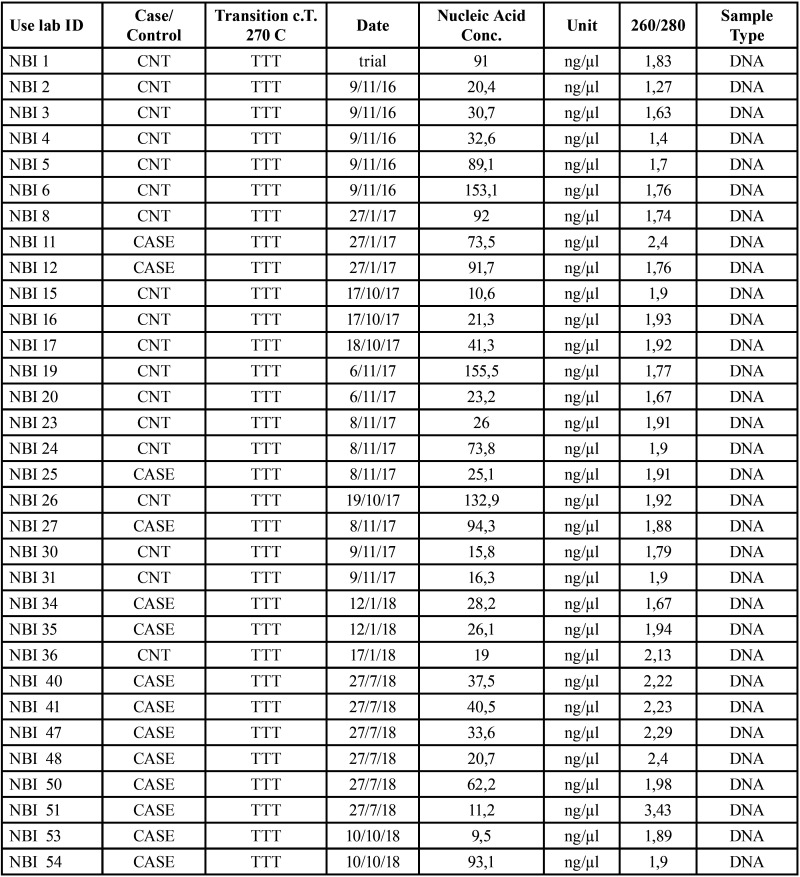
First quantification of the samples selected for the study. Case / control designation was made according to what the clinician who performed the biopsies dictated. (TTT - Transition c.T270C) exon 5 of the p53 gene as a transition from a T to C base in codon 270.

All patients who participated in the study were checked within one week after biopsy and provided with biopsy results. Based on this result, the patient underwent the removal of the lesion or its follow-up, according to the therapeutic indication of the current protocols.

-p53 Genetic study

Tissue sections were made from very small samples, depending on the size of the tissue, between 10-20 sections of 10 microns were made.

The extraction was carried out according to the GeneReadTM DNA FFPE protocol ref.180134 (formalin-fixed paraffin embedded) of QIAGEN. The first part was done manually until the paraffin dissolution, and from step 13 of the protocol, it is automated on the QIACUBE equipment of the QIAGEN brand.

DNA extractions were quantified on a Thermo Nanodrop 2000C spectrophotometer to check the quantity and quality of DNA.

The purpose was to use DNA to search our population for point mutations described in the reference article (14), which are located between the exon 5 to 8 regions of the P53 gene.

The article describes a point mutation in exon 5 of the P53 gene as a base transition T to C at codon 270 (c.T 270 C). The amplification of the DNA region where we are looking for the mutation is done by polymerase chain reaction (PCR) with the BIOTAQ DNA Pol Kit, Ref. BIO-2104040 500 from Bioline.

The design of the oligonucleotide sequences of the specific region of the gene that we want to amplify is carried out with the help of the Software Primer3 Output V.4. This software ensures that we are amplifying the region where the point mutation we want to study is located.

The Oligos sequences are:Left (reverse) exon 5 P53 / 20mer / 0,0250 UMO / Desalted Purification GACAAGGGTGGTTGGGAGTA (5`-3`) SIGM A Aldrich.

Right (forward) exon 5 P53 / 20mer / 0,0250 UMO / Desalted Purification TCTTGCGGAGATTCTCTTCC (5`-3`) SIGM A Aldrich.

The temperature of the primers was 60 ° C.

The PCR conditions are a hybridization temperature of 60°C for one minute, for an amplicon size of 221 base pairs. The size of the amplified DNA fragment is confirmed by electrophoresis on a 2% agarose gel.

Prior to sequencing, the PCR samples were purified using the INVITROGEN ExoSAP-IT For PCR Product Cleanup kit. 78201 1 ML.

The sequencing of the samples was performed on the Applied Biosystems 3500 Genetic Analyzer sequencer and the sequences were analyzed using the CHROMAS software version.2.5.1.

Since no patient in our sample population from southern Spain had the point mutation (exon 5 of the p53 gene as a transition from a T to C base in codon 270 (c.T270C)) described in the article that was taken as a reference (14), a search for mutations in the complete exons 6, 7 and 8, also described in the article by S. Tang *et al*. (14), was considered, with all probability that it would give the same result in our population.

In databases such as the International Agency for Research on Cancer (IARC) we observed that the exon sizes of 5 and 6 as well as 8 and 9 can be tracked complete, as they have amplicon sizes of 476 and 445 base pairs, respectively. This allowed us to be more rigorous given the small cohort of patients.

The methodology is similar to that followed previously in the search for the described point mutation. The analysis is done from sequences in both directions to rule out false positives.

The Oligos sequences for the full EXON 8 and 9 P53 GEN are:

Left (reverse) exon 8-9 P53 / 20mer / 0,0250 UMO / Desalted Purification TTGGGAGTAGATGGAGCCT(5`-3`) SIGM A Aldrich. The temperature of the primers was 61.2 ° C 

Right (forward) exon 8-9 P53 / 20mer / 0,0250 UMO / Desalted Purification AGTGTTAGACTGGAAACTTT (5`-3`) SIGM A Aldrich. The temperature of the primers was 51.9 ° C.

The PCR conditions are a hybridization temperature of 55°C for 45 seconds, for an amplicon size of 445 base pairs.

The Oligos sequences for full EXON 5 and 6 P53 GEN are:

Left (reverse) exon 5-6 P53 / 20mer / 0,0250 UMO / Desalted Purification TGTTCACTTGTGCCCTGACT(5`-3`) SIGM A Aldrich. The primer temperature was 63.5°C 

Right (forward) exon 5-6 P53 / 20mer / 0,0250 UMO / Desalted Purification TTAACCCCTCCTCCCAGAGA (5`-3`) SIGM A Aldrich. The temperature of the primers was 64.7°C.

The PCR conditions are a hybridisation temperature of 58ºC for 30 seconds, for an amplicon size of 476 base pairs.

Prior to sequencing, the PCR samples were purified using the ExoSAP-IT for PCR Product Cleanup kit from INVITROGEN ref. 78201 1 ML.

The sequencing of the samples is carried out on the Applied Biosystems 3500 Genetic Analyzer sequencer, and the sequences are analysed using the Software Code Aligner. This software looks for any differences from the original sequence.

Only, patient NBI57 presented a change in the triplet TAT for ATT in EXON 6, the change in the nucleotide T for A and in both directions, Foward and Reverse. The exact location according to NCBI is GRCh37p13 on chromosome 17, EXON 6 of the p53 gene and the change is in the nucleotide C.613T>A; NM_000546.

This result was described in a previously published paper by our group (15).

-NOTCH1 Genetic study

Since only patient NBI57 had a mutation, it was decided to rework the samples to search for new mutations, including those described for NOTCH1.

A genetic study of biopsied lesions was carried out by direct sequencing of amplified DNA products extracted from biopsies to analyze the presence of NOTCH1 gene mutations as an important biomarker for the detection of oral mucosal lesions and the prediction of their malignant progression.

DNA extraction was carried out through preprocessing of the sample dewaxing carried out with the use of the Minikit QIAamp DNA FFPE Tissue Reference 56404 extraction kit of the QIAGEN brand in the QIAcube automated station of the same brand.

Samples were kept in deep freeze until the end of the study, creating a database with parameters of interest, such as ratios and concentrations of these, with the help of the Nanodrop 2000C spectrophotometer.

With the DNA of the samples, 4 amplification reactions were performed using the polymerase enzyme (PCR reaction).

Before sequencing of the samples, the purification of the samples was performed using the ExoSAP-IT for PCR product cleanup kit of the INVITROGEN brand ref. 78201 1 ML.

To detect somatic mutations in NOTCH1 in our samples, we worked with a non-customised panel of mutations present in other cancers for NOCTH1 (Table 2) and used the software TaqMan Mutation Detection Assays.

**Table 2:**
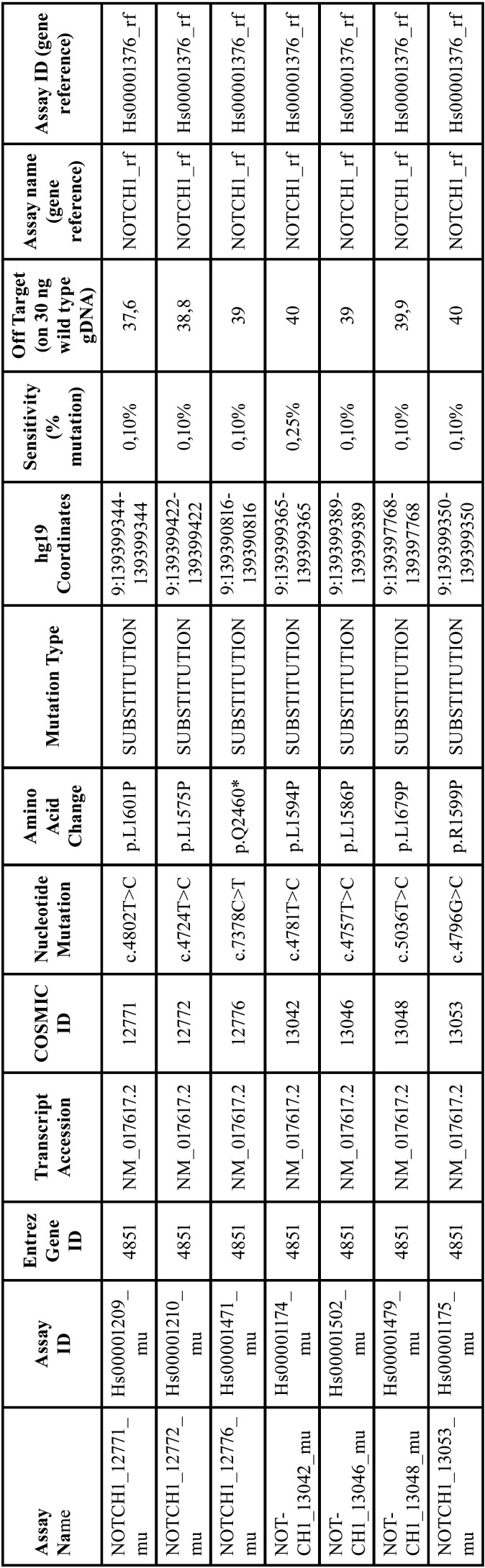
Panel of mutations described for NOTCH1 showing the gene where the mutation is located, as well as the amino acid change, type of mutation, and coordinates.

TaqMan mutation detection assays work with castPCR technology, which refers to TaqMan PCR® specific to competitive alleles. The technology used is very specific and sensitive and can detect and quantify rare amounts of mutated DNA in a sample containing large amounts of normal wild-type gDNA. Our study is a mutant allele trial.

After the extraction and purification of gDNA, it was quantified. All samples were normalized to 10gr/l and to a final volume of 10l. (Table 3).

**Table 3:**
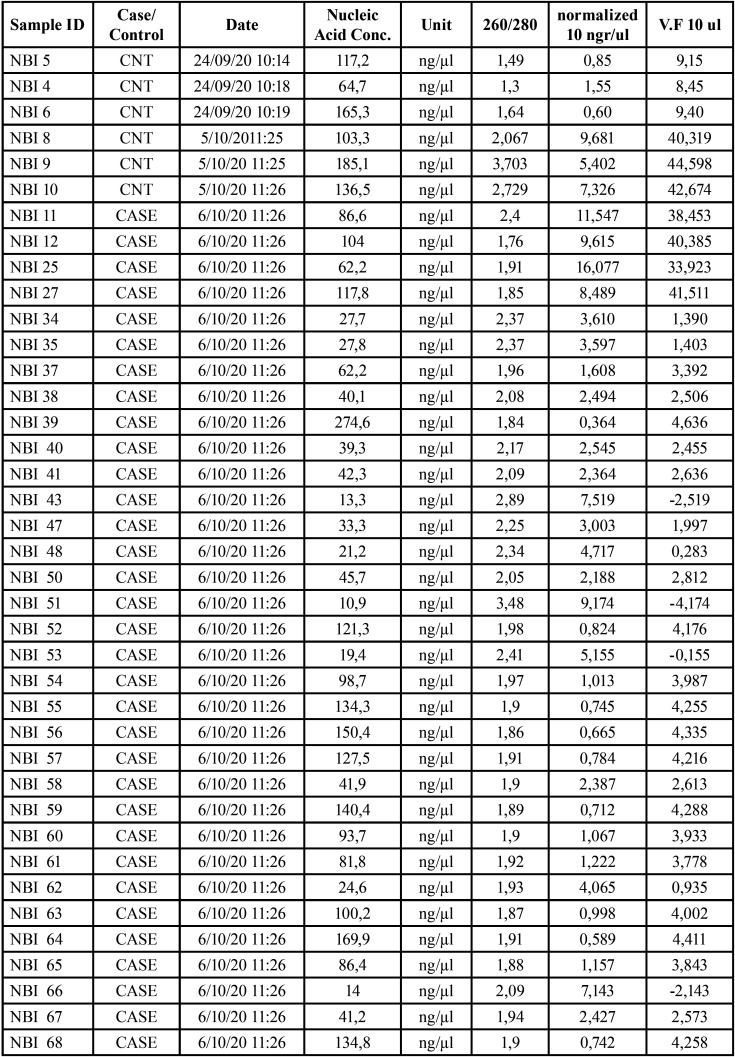
Amounts of extracted, purified, and normalized DNA per sample. Case / control designation was made according to what the clinician who performed the biopsies dictated.

For the analysis of the mutations, the Mutation Detector Software was used, which includes the mutation probes and other reference probes for NOTCH1. To determine the mutation in this software, 3 positive controls that are in very good condition are selected and placed in triplicate to ensure that the cut-off value (CT) is between 18 and 28. In our case, selecting CT samples that are between 18 and 28 was the difficult thing, given that our samples are made of paraffin and therefore their quality has decreased, although even so, 3 controls were achieved in good condition.

In the software, the control samples are those where the researcher does not expect to find any mutations. These samples are selected in the Control column of the software as POSITIVE. However, samples considered mutants will be selected as NEGATIVE and correspond to probes from all regions of the NOTCH1 gene that have already described mutations.

The first thing that software needs to do is determine the cutoff value. The cutoff is an arithmetic performed by the software that reports a number that the higher it is, the more certain it is that the mutation is present or not in our samples. What the software does is a ΔCT considering the CT of the allele mutant and the CT of the reference gene.

Deciding which cut-off value, the software needs to detect the mutation, is only done with positive controls.

Our ΔCT calculation is 4.27. It cannot be adjusted further, as the samples are in paraffin, yet the result is valid.

## Results

-Participants and Characteristics of the Lesions

A total of 22 patients were recruited between November 2017 and May 2019 who showed potentially malignant oral mucosal lesions. Of the 22 patients recruited, 9 were women and 13 were men. Of these, 10 patients were under the age of 60 and 12 patients were over the age of 60. Regarding a history of oral cancer, only 3 patients had suffered previous oral carcinoma and none of them had received radiation therapy.

Regarding the general health status of the patients, 19 of them were healthy and 3 had systemic diseases.

As for habits:

• Alcohol consumption: 17 nondrinkers, 4 drinkers and 1 former drinker.

• Tobacco: 8 nonsmokers, 4 former smokers, 6 who smoked less than one pack a day, and 4 who smoked more than one pack a day.

• Oral hygiene and tooth brushing: 5 patients who brushed 3 times a day, 15 who brushed once a day and 2 patients who brushed less than seven times a week.

Regarding the location of injuries:

• Retromolar trine: 1

• Buccal mucosa: 4

• Ventrolateral tongue: 7

• Lip mucosa: 1

• Floor of the mouth: 3

• Dorsal tongue: 2

• Attached gum: 1

• Palate: 3

Regarding the clinical diagnosis of lesions.

• 11 leukoplakias

• 4 erythroplakias

• 5 leukoerythroplakias

• 2 ulcerative lesions.

Lesion size.

• < 1cm: 5 lesions

• 1-3 cm: 14 lesions

• > 3 cm: 3 lesions

Other features:

• Induration: 5 lesions

• Spontaneous pain: 5 injuries

• More than 850 days of progression: 4 lesions

Pathology.

• Low-grade dysplasia: 15

• High-grade dysplasia: 2

• Carcinoma 5

-NOTCH1 Analysis

The advantage of using the Mutation Detector software is that it is a software that facilitates the detection of the mutation, the greater the amount of DNA, even though the mutation has very little presence.

In our study, we work with 20ngr total, able to detect mutations that are in a 0.1% presence.

Remember that the cut-off value that the software needs to detect the mutation is only done with positive controls. In our samples, a cutoff ΔCT detection calculation of 4.27 is obtained.

Given that our samples have a ΔCT value greater than the CT detection ΔCT cutoff value (4.27), the software classifies gDNA samples as mutation not detected. The samples are mutation negative or below the limit of detection for the TaqMan Mutation Detection Assays.

## Discussion

In exploring the genetic origins of head and neck squamous cell carcinoma, Agrawal *et al*. in 2011 were the first to identify mutations in NOTCH1 and suggested that nearly 40% of the 28 mutations identified in that gene would impede the product of the gene, so they proposed that NOTCH1 could function as a tumor suppressor gene instead of a proto-oncogene (13).

Since then, its role in the carcinogenesis of HNSCC and, above all, OCSCC has been controversial, as studies reveal that NOTCH1 could perform various functions as both an oncogene and as a tumor suppressor gene (1,3-10,12). Similarly, improved and worse survival has been observed in HNSCC patients with alterations in NOTCH1 (3).

In our sample of 22 patients, 15 with low-grade dysplasia, 2 with high-grade dysplasia, and 5 with carcinoma, the NOTCH1 mutation is not detected, so the sample studied does not present the mutation or is below the limits of software detection.

In the study by Song *et al*. (16), they found higher rates of NOTCH1 mutation (43%) in Chinese patients, while mutations for a Singaporean population with squamous cell carcinoma in the tongue were not common, concluding that inactivation of the NOTCH1 gene may play complex genetic interactions in OCSCC carcinogenesis (1,3,4,6,10). Similarly, the study highlights that this mutation frequency found in the Chinese population is significantly higher than that found in the Caucasian population (10-15%) (1,3,4). Therefore, other studies report that NOTCH1 is a tumor suppressor gene that finds gene mutations in 54% of oral squamous cell carcinoma and 60% of preneoplastic lesions in Chinese patients (13). All this suggests that NOTCH1 mutations play a role in the carcinogenicity of OCSCC, and this is confirmed by another study in a Taiwanese population together with the previous study in the Chinese population (1).

However, it should be considered that these mutations may be caused by different etiological factors and that this is one of the possible explanations for the difference in prevalence in terms of the NOTCH1 mutation from one population to another, such as, for example, the genetic background of the population (3).

On the other hand, observations on the role of NOTCH1 as an oncogene or as a tumor suppressor gene assume different functions in carcinogenesis and, therefore, that there must be other interactions within the tumor microenvironment that influence or alter the expression of this gene (7). Therefore, another article attempts to explain it (3), referring to the fact that the reason for these differences can be found in the cellular context, as the NOTCH pathway plays opposite roles in malignant lesions depending on the type of cell, tissue, level of expression, and interaction with other pathways.

Some studies associate NOTCH1 expression with advanced clinical stages and nodal metastases (9,12). However, the authors of the study concluded that NOTCH1 expression did not influence the prognosis of patients with oral cancer, the results of other authors (12), although it appears to contribute to the identification of patients with OSCC.

In the study by Kujan *et al*. (7) they warn that the level of expression for NOTCH1 was reduced from nondysplastic tissue to OSCC, although these results were not statistically significant, indicating that these observations may propose that NOTCH1 expression is not a good indicator of OSCC development (3,7). Other studies documented in this article (7) and others support this view, as they demonstrated that neither the expression of NOTCH1 nor its translocation is observed in their cases of OSCC.

In another study (8), whose objective was to determine the expression of biomarkers, among them NOTCH1, in stem cells in layers of dysplastic oral epithelium, they obtained as a result that the SIGNAL of NOTCH1 was weak and highly variable, so it does not differ appreciably in the test tissues compared to the control tissues, so they concluded that NOTCH1 is not a good biomarker.

Another study that wanted to know the immunoexpression of NOTCH1 in four subtypes of oral cancer (well-differentiated OSCCs, poorly differentiated OSCCs, verrucous carcinomas and basaloid squamous cell carcinoma (BSCCs)) did not show significant differences (12). These results were supported by other studies (17-20).

However, since the NOTCH1 mutation has been found with high frequency in HNSCC and is nevertheless rare in cancers other than SCC (4), the pathogenesis must be different, as stated above, influenced by the tumor context in which it develops.

In summary, the action of NOTCH1 in the development of squamous cell carcinomas, its role as both a tumor suppressor gene and as a protooncogene, is not yet fully understood. It appears that the activity that NOTCH develops as a tumor suppressor is closely related to the physiological function of NOTCH in the development and maintenance of the squamous epithelium (7), the loss of NOTCH1 produces changes in the signaling of other molecules that result in decreased cell differentiation, as well as an increase in the number of keratinocytes, variations in cellular senescence, and altered epithelial integrity (9). Although its oncogenic role promotes cell proliferation, inhibition of apoptosis, promotion of TMS, stem cell reserve, and angiogenesis, among other alterations (9).

## Conclusions

The study in our cohort of 22 patients diagnosed with potentially malignant disorders and/or carcinomas starts from the idea of knowing the presence of the point mutation (exon 5 of the p53 gene as a transition from a T to a C base at codon 270 (c.T270C)) described in the article by S. Tang (14), which was taken as a reference for the first article published in this sample of patients (15). As no patient had this mutation, it was then decided to search the entire exons 6, 7, and 8 for the p53 gene. Given that only patient NBI57 presented a change in the triplet of TAT for ATT in EXON 6, the change in the nucleotide T for A and in both forward and reverse directions, a search for new mutations was considered, including NOTCH1, as there are articles verifying a high mutation of this gene in OSCC and in potentially malignant lesions with high- and low-grade dysplasia (4). However, again, in our sample, no mutation for NOTCH1 was observed.

Although there is no consensus, except for the large variability in mutational influence, and given that in our sample of patients where 15 lesions with high-grade dysplasia, 2 lesions with high-grade dysplasia, and 5 carcinomas were diagnosed, no mutation for NOTCH1 was observed, it is concluded that further studies are required to understand not only the role of NOTCH1 in carcinogenesis, but also how it can act completely oppositely in the development and progression of HNSCC, in our case OSCC, as well as its signaling within the tumor microenvironment.

Likewise, the great genetic variability of oral cancer is highlighted, as well as the difference in the prevalence of mutations in different populations. However, given our sample of 22 patients, we cannot conclude that NOTCH1 is not useful in the southern Spanish population.

-Limitations of the study

The main limitation of the study is the small sample of patients (22 patients in total), so the usefulness of NOTCH1 as a molecular biomarker to predict the risk of malignancy of precancerous lesions in our sample of patients from southern Spain cannot be verified. To know whether NOTCH1 mutations are present in patients from southern Spain diagnosed with premalignant lesions and/or carcinoma, the sample of patients would have to be increased in a sufficiently representative number to reach general conclusions about the population.
